# Hamstring Tendon vs. Bone-Patellar Tendon-Bone Grafts for Primary Anterior Cruciate Ligament Reconstruction in Football (Soccer) Players: A Systematic Review and Meta-Analysis

**DOI:** 10.7759/cureus.100912

**Published:** 2026-01-06

**Authors:** Kyle Muscat, Jordan Calleja

**Affiliations:** 1 General Surgery, Mater Dei Hospital, Msida, MLT

**Keywords:** acl reconstruction, donor-site morbidity, football, graft failure, hamstring tendon, knee stability, patellar tendon, return to sport, soccer

## Abstract

Anterior cruciate ligament (ACL) rupture is a common and career-impacting injury among football players, often requiring surgical reconstruction. The two most commonly used grafts, hamstring tendon (HT) and bone-patellar tendon-bone (BPTB), have both shown favorable outcomes, but the optimal choice remains debated. This systematic review aimed to evaluate and compare outcomes of ACL reconstruction using HT vs. BPTB autografts specifically in football players. A systematic literature search was conducted across PubMed, Cochrane Library, and MEDLINE databases from inception to January 2025. Eligible studies included randomized controlled trials and observational cohort or case-control studies comparing HT and BPTB autografts in football players undergoing primary ACL reconstruction. The primary outcome was graft failure rate. Secondary outcomes included return to sport, isokinetic muscle strength, patient-reported outcome measures (PROMs), donor-site morbidity, and knee stability. Meta-analyses were performed for outcomes reported in ≥2 studies with statistical heterogeneity (I²) ≤ 60%. Outcomes with high heterogeneity or limited reporting were summarized narratively using the Synthesis Without Meta-analysis (SWiM) guidelines. Risk of bias was assessed using the Risk of Bias 2 (RoB2) and Risk of Bias in Non-Randomized Studies of Interventions Version 2 (ROBINS-I V2) tools. Nine studies with 1,173 participants met the inclusion criteria. Meta-analysis of five studies (n = 1,013) showed no statistically significant difference in graft failure rates between the graft types (HT: 7.88%, BPTB: 6.34%, OR = 1.46, 95% CI: 0.80-2.67, *p* = 0.22). Return-to-sport rates were also comparable between graft types. Evidence for secondary outcomes was limited. Meta-analysis of extensor strength, based on two studies and heavily weighted toward a single trial, suggested greater strength following HT reconstruction, while flexor strength and donor-site morbidity findings were derived from single studies. PROMs and knee stability outcomes showed no consistent differences. Risk of bias was moderate to serious in most observational studies. This systematic review and meta-analysis found no clear superiority between HT and BPTB grafts in terms of failure rates, return to sport, or stability outcomes in football players. In contrast, evidence regarding muscle strength recovery and donor-site morbidity remains limited and preliminary. These secondary findings should be interpreted cautiously, highlighting the need for further high-quality, football-specific trials.

## Introduction and background

The anterior cruciate ligament (ACL) is the most commonly injured knee ligament, particularly among athletes involved in high-demand sports such as football [1]. The UK National Ligament Registry reported that 47.8% of ACL injuries in 2022 occurred while playing football [2]. For football players, ACL injury is considered career-threatening due to its association with prolonged rehabilitation, difficulty returning to pre-injury performance levels, and an increased risk of re-injury [3-5].

ACL reconstruction remains the gold standard treatment for ACL rupture in football players [4], restoring knee stability and enabling return to play. Grafts used for reconstruction are typically autografts or allografts, with hamstring tendon (HT) and bone-patellar tendon-bone (BPTB) autografts being the two most widely used due to their favorable outcomes and surgical familiarity [6,7]. However, the optimal graft choice for football players remains widely debated.

Large-scale observational studies have reported higher revision rates following ACL reconstruction with HT grafts, particularly in younger populations [8-10]. In contrast, meta-analyses of randomized controlled trials (RCTs) in mixed athletic populations have demonstrated no significant difference in graft failure rates between HT and BPTB grafts [11,12]. These conflicting findings highlight uncertainty regarding whether graft choice meaningfully influences outcomes in football-specific populations.

Beyond graft survival, other clinically relevant outcomes, including return-to-sport rates, isokinetic muscle strength recovery, patient-reported outcome measures (PROMs), donor-site morbidity, and postoperative knee stability, are critical determinants of successful return to football. These factors may differ according to graft type and can influence both performance and long-term knee health. However, evidence regarding these outcomes remains inconsistent and has not been systematically synthesized in football players specifically [6,10-15].

Therefore, the aim of this systematic review and meta-analysis was to evaluate and compare the clinical outcomes of HT and BPTB autografts for ACL reconstruction in football players, with graft failure rates as the primary outcome.

## Review

Methodology

Review Registration and Reporting

This systematic review was conducted following the Preferred Reporting Items for Systematic reviews and Meta-Analyses (PRISMA) 2020 and Synthesis Without Meta-analysis (SWiM) guidance [16,17]. Its protocol was prospectively registered on PROSPERO (CRD42024619723).

Inclusion and Exclusion Criteria

Eligibility criteria followed a PICOS framework (Table 1). Included studies were RCTs or prospective and retrospective cohort and case-control studies that compared ACL reconstruction using HT vs. BPTB graft in football players undergoing primary unilateral ACL reconstruction. Additionally, studies had to report at least one of the specified primary or secondary outcomes to be included in the systematic review. Studies were excluded if they were cross-sectional, case reports, or non-observational studies; involved patients with previous ACL injuries or multi-ligamentous knee injuries; or were published in a language other than English.

**Table 1:** PICOS framework ACL, anterior cruciate ligament; BPTB, bone-patellar tendon-bone; HT, hamstring tendon; PICOS, Population, Intervention, Control, Outcome, Study design; PROMs, patient-reported outcome measures; RCT, randomized controlled trial

PICOS	Inclusion criteria	Exclusion criteria
Population	Football players who underwent primary unilateral ACL reconstruction	Previous ACL injury, bilateral ACL injury, and multi-ligamentous knee injury
Intervention	ACL reconstruction using HT graft	Revision ACL reconstruction using HT graft
Control	ACL reconstruction using BPTB graft	Revision ACL reconstruction using BPTB graft
Outcome	Primary: graft failure rates; secondary: return-to-sport rate, isokinetic knee flexor and extensor strength, PROMs, donor-site morbidity, and knee stability	-
Study design	RCTs, prospective and retrospective observational cohorts, and case-control studies	Cross-sectional studies, case reports, case studies, editorials, and expert opinions
Language	English	Non-English
Time frame	From inception to the date of search	-

Data Sources and Search Strategies

A systematic literature search was conducted across PubMed, Cochrane Library, and MEDLINE (via Ovid) databases from inception to January 20, 2025.

The search strategy was guided by the PICOS framework. A detailed search strategy for each database is provided in Appendix A, Appendix B, and Appendix C. The search was supplemented with manual screening of reference lists of relevant studies.

Data Extraction and Management

Data were extracted manually by two independent reviewers using a data extraction template on Covidence. Extracted data included information about the study: main author, year of publication, study title, study setting, sponsorship source, study design, sampling technique, aims and objectives, and reported outcomes. Information about the participants included inclusion and exclusion criteria, sample size, number and reasons for withdrawal, mean age, sex, level of competition, injured side, and body mass index. Information about the intervention and control included number of participants allocated, surgical technique, rehabilitation protocol, and time from injury to surgery. An online Google Sheets document (Google LLC, Mountain View, California, USA) was then created for data management.

Types of Outcomes

The primary outcome of this study was the graft failure rate following ACL reconstruction using HT vs. BPTB graft. Secondary outcomes were return-to-sport rates and level of return, time to return to sport, isokinetic knee flexor and extensor strength, Lysholm score, Tegner score, anterior knee pain, hamstring tendonitis, patellar tendonitis, Lachman’s grade, and anterior drawer test grade.

Data Analysis

Meta-analyses were conducted using RevMan for outcomes reported in at least two studies, provided that heterogeneity (I²) was ≤ 60%. Heterogeneity was assessed using the I² statistical test. We acknowledged that a heterogeneity cutoff of 60% may include moderate heterogeneity when considering the results. A random effects model was chosen, assuming high heterogeneity amongst included studies. The restricted maximum likelihood method was used to estimate the heterogeneity variance (τ²). Statistical significance was defined as a p-value < 0.05.

A meta-analysis was performed for the primary outcome, graft failure rate, using data from all five eligible studies. ORs with 95% CIs were used as the summary effect measure. Additional meta-analyses were conducted for secondary outcomes reported in at least two studies. Binary outcomes were pooled using ORs with 95% CIs, while continuous outcomes were analyzed using either standardized mean differences or mean differences as appropriate. Forest plots were generated in RevMan to visually present effect estimates.

For outcomes with high heterogeneity (I² > 60%), specifically isokinetic flexor muscle strength and Tegner score, meta-analysis was not conducted. A narrative synthesis following the SWiM guidelines was instead performed. Data were also presented using forest plots without pooled estimates.

Risk of Bias Assessment

The Cochrane Risk of Bias 2 (RoB2) tool was used to assess the risk of bias in all included RCTs [18]. For observational studies, the Risk of Bias in Non-Randomized Studies of Interventions Version 2 (ROBINS-I V2) tool was applied [19]. Due to the limited number of studies included in the meta-analyses, publication bias was not assessed statistically.

Results

Included Studies

The initial search was completed in January 2025 and identified a total of 160 articles (PubMed = 61, Cochrane Library = 49, MEDLINE = 48, and citation searches = 2). All articles were exported to EndNote Reference Manager, where 39 duplicates were automatically removed. Following title and abstract screening, 35 articles were retrieved for full-text review. After applying the inclusion and exclusion criteria, nine studies met eligibility and were included in the systematic review.

**Figure 1:**
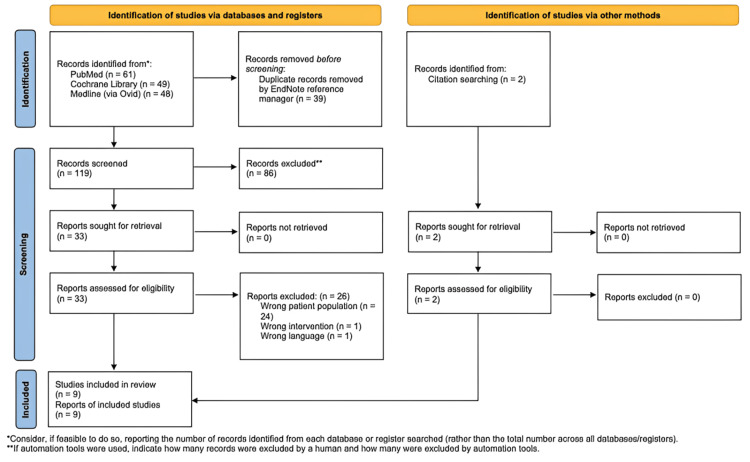
Study selection process: PRISMA flow diagram PRISMA, Preferred Reporting Items for Systematic reviews and Meta-Analyses

The study selection process is outlined in the PRISMA flow diagram (Figure 1).

Characteristics of Included Studies

Nine studies were included, with a total sample size of 1,173 participants. Of these, two were RCTs [6,20], and seven were observational studies [10,13,15,21-24]. Follow-up times varied across studies, ranging from six months to 10 years.

A total of 845 participants underwent ACL reconstruction using an HT autograft, whilst 328 received BPTB autografts. Where reported, the mean age of participants in the HT group ranged from 15.4 ± 1.3 to 29.1 ± 6.7 years, and from 15.4 ± 1.3 to 32.6 ± 4.0 years in the BPTB group.

Three studies included only male football players [20,22,23], while five studies included both male and female football players [6,10,13,15,24]. Britt et al. was the only study to include only female football players [21]. The male:female ratio, where reported, was 563:343 across all studies, with 64:42 in the HT group and 67:49 in the BPTB group, where reported.

The level of competition varied across the studies. Two studies included players playing across all levels, including the recreational level [10,24]. Four studies included only players who were playing at a competitive level [6,13,15,22]. Three studies did not report the participants’ competition level [20,21,23].

All included studies compared clinical outcomes between ACL reconstruction with HT and BPTB grafts. The primary outcome, graft failure rate, was reported in five studies [6,10,13,21,24]. Secondary outcomes, including return to sport, isokinetic muscle strength, PROMs (Lysholm and Tegner scores), donor-site morbidity (anterior knee pain, patellar tendonitis, and hamstring tendonitis), and knee stability outcomes were variably reported across the studies.

Surgical technique and rehabilitation protocols were described in all studies except those of Sandon et al. and Walden et al. The surgical techniques were comparable for both HT and BPTB graft groups, and postoperative rehabilitation programs were identical for both intervention groups within each study.

A summary of key characteristics of the included studies can be found in Table 2.

**Table 2:** Characteristics of included studies ACL, anterior cruciate ligament; ACLR, anterior cruciate ligament reconstruction; BPTB, bone-patellar tendon-bone; CERS, Centre for Sports Rehabilitation; HT, hamstring tendon; RCT, randomized controlled trial; SNKLR, Swedish National Knee Ligament Registry

Author and year	Country	Study design	Study participants	Sample size	Level of competition	Interventions	Rehabilitation	Outcomes reported	Follow-up period
Guglielmetti et al. (2021) [6]	Brazil	RCT	Professional or semi-professional football players	62	Semi-professional or professional (4+ sessions/week)	HT vs. BPTB	Both groups of patients underwent the same institutional timeline-based physical therapy protocol	Graft failure rate, return to sport, anterior knee pain, hamstring tendonitis, patellar tendonitis, Lachman grade, anterior drawer test, Lysholm score	2 years
Laboute et al. (2018) [10]	France	Non-randomized retrospective cohort study	Athletes who had undergone an ACL autograft reconstruction and who received rehabilitation care at the European CERS	153 football athletes (955 total)	28 international (2.9%), 319 national (33.4%), 470 regional (49.2%), 138 recreational (14.5%)	HT vs. BPTB	Rehabilitation care at the European CERS	Graft failure rate	2 years
Laboute et al. (2010) [13]	France	Non-randomized retrospective cohort study	Sportspeople playing at a regional or higher level	53 football athletes (298 total)	Regional or higher (national or international)	HT vs. BPTB	Rehabilitation care at the European CERS	Graft failure rate	4 years
Waldén et al. (2010) [15]	Sweden	Non-randomized prospective cohort study	Football players playing in European professional men’s first leagues and those playing in Swedish men’s and women’s first leagues	71	Professional	HT vs. BPTB	Not reported	Return to sport (time)	8 years
Mohammadi et al. (2013) [20]	Iran	RCT	Soccer players	42	Not reported	HT vs. BPTB	Accelerated rehabilitation protocol in a unique rehabilitation center	Isokinetic knee flexor strength, isokinetic knee extensor strength	8 months
Britt et al. (2020) [21]	USA	Non-randomized retrospective cohort study	Skeletally mature adolescent female soccer players	71	Not reported	HT vs. BPTB	Postoperatively, all patients followed the same rehabilitation protocol and restrictions, with return to play between 9 and 12 months	Graft failure rate, return to sport, Lysholm score, Tegner score	2 years
Milutinovic et al. 2023 [22]	Serbia	Non-randomized prospective cohort study	Soccer players competing at the international level (elite)	17	International level (elite)	HT vs. BPTB	All subjects underwent the same standardized 6-month rehabilitation program at the same private physiotherapy center	Isokinetic knee flexor strength, isokinetic knee extensor strength	6 months
Rudroff (2003) [23]	Germany	Randomized prospective cohort study	Male soccer players	30	Not reported	HT vs. BPTB	6-week rehabilitation program in a sports medicine clinic	Isokinetic knee flexor strength, isokinetic knee extensor strength, Lysholm score, Tegner score	2 years
Sandon et al. (2020) [24]	Sweden	Non-randomized case-control study	Soccer players in the SNKLR who underwent primary ACLR	684	Recreational (67), lower divisions (317), middle divisions (179), higher divisions (26), top division (7), national team (1), youth (87)	HT vs. BPTB	Not reported	Graft failure rate	10 years

Graft Failure

**Figure 2:**
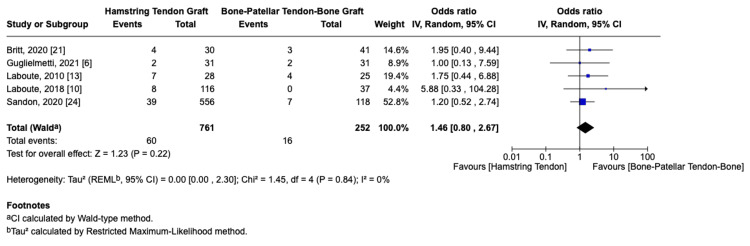
Graft failure rate forest plot

Five studies [6,10,13,21,24] reported data on graft failure rates and were included in the meta-analysis. The pooled analysis comprised 761 participants in the HT group and 252 in the BPTB group. The meta-analysis revealed no statistically significant difference in graft failure rates between the two graft types, with 7.88% vs. 6.34% graft failure rates in the HT vs. BPTB arms, respectively (OR = 1.46, 95% CI 0.80-2.67, p = 0.22, Figure 2). Statistical heterogeneity was low (I² = 0%).

Among the included studies, Sandon et al. provided the longest follow-up duration at 10 years postoperatively, with failure rates of 7% in the HT group and 5.9% in the BPTB group (p = 0.67) [24]. Guglielmetti et al., the only RCT, reported equal failure rates of two out of 31 patients in both groups (p = 0.99) [6].

Return to Sport

Three studies [6,15,21] reported outcomes related to return to sport. Two of the studies [6,21] provided data on both the rate of return and the level at which players returned. A total of 133 football players were included across the two studies: 61 in the HT group and 72 in the BPTB group.

**Figure 3:**
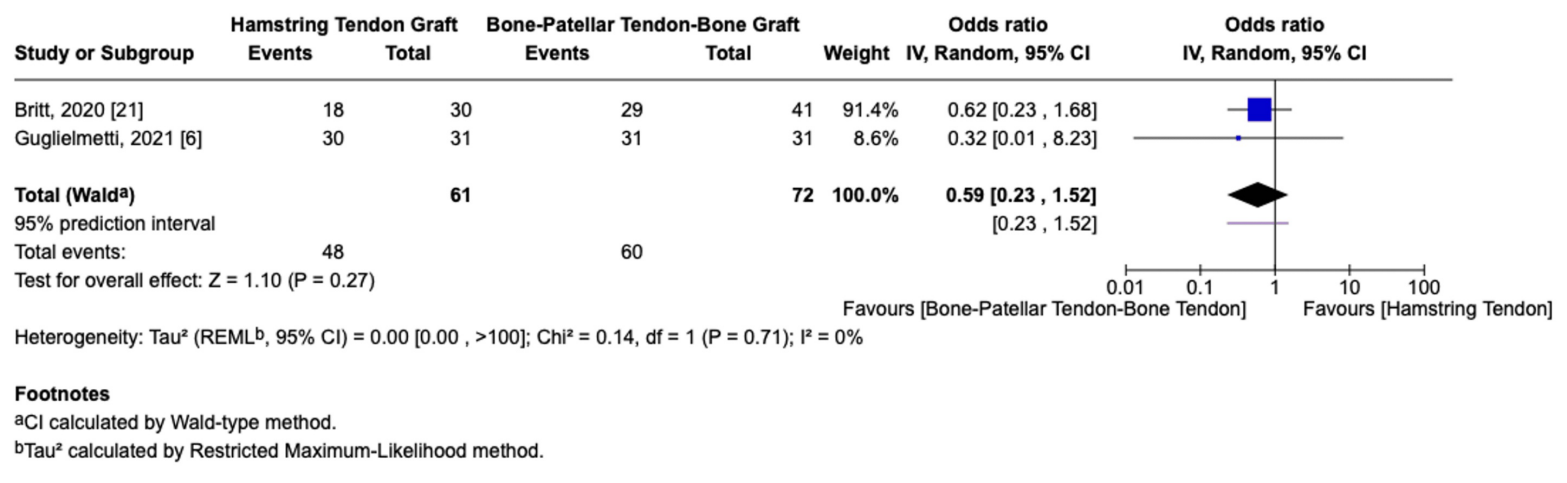
Return-to-sport rate forest plot

The pooled return-to-sport rate was 78.7% in the HT group and 83.3% in the BPTB group. Meta-analysis showed no statistically significant difference between the graft types (OR = 0.59, 95% CI: 0.23-1.52, p = 0.27, I² = 0%, Figure 3).

Regarding return to pre-injury level, Guglielmetti et al. reported comparable rates between groups (HT: 54.8%, BPTB: 51.6%) [6], while Britt et al. found a higher return in the BPTB group (41.5% vs. 30%) [21], but neither study found statistically significant differences (p = 0.71 and p = 0.40, respectively).

The third study, by Walden et al., examined time to return to sport. They observed shorter mean times in the HT group for both return to training (190.9 ± 46.4 days vs. 216.9 ± 82.9 days) and return to match play (227.5 ± 58.6 days vs. 253.8 ± 93.3 days), though these findings were not statistically significant (p = 0.18 and p = 0.12, respectively) [15].

Postoperative Isokinetic Muscle Strength

Three studies [20,22,23] reported data on postoperative isokinetic flexor muscle strength, including a total of 89 football players: 45 in the HT group and 44 in the BPTB group. Due to substantial statistical heterogeneity (I² = 96%), a meta-analysis was not conducted.

**Figure 4:**
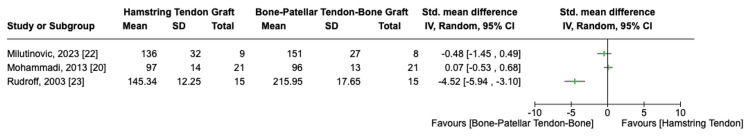
Isokinetic flexor muscle strength forest plot

Milutinovic et al. and Mohammadi et al. found no significant difference at six to eight months [20,22]. Rudroff reported significantly greater isokinetic flexor muscle strength in the BPTB group at two years (p < 0.05) [23]. These findings are visually presented in Figure 4.

**Figure 5:**
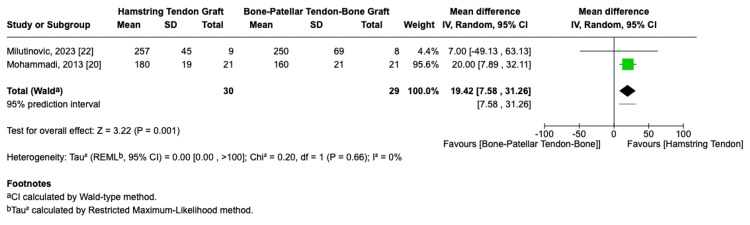
Isokinetic extensor muscle strength forest plot

Two of the studies [20,22] reported on postoperative isokinetic extensor muscle strength. A meta-analysis of these studies demonstrated a statistically significant advantage for HT autografts, with an MD of 19.42 (95% CI: 7.58-31.26, p = 0.001, I² = 0%, Figure 5).

PROMs: Lysholm Knee Score and Tegner Activity Score

Three of the included studies reported data on postoperative Lysholm Knee Score [6,21,23], while two studies additionally reported postoperative Tegner Activity Score [21,23]. All studies assessed these PROMs at two years post-surgery.

**Figure 6:**
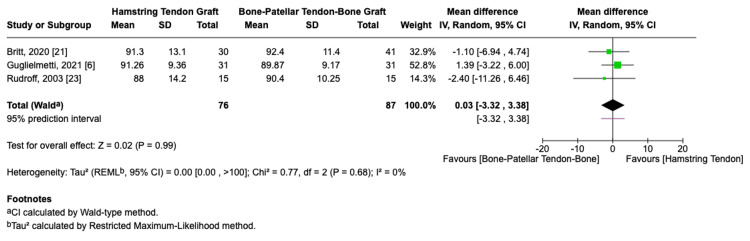
Lysholm knee score forest plot

A meta-analysis of the Lysholm Knee Score, involving a total of 163 football players (76 in the HT group and 87 in the BPTB group), showed no statistically significant difference between the two graft types with an MD of 0.03 (95% CI: -3.32-3.38, p = 0.99, I² = 0%, Figure 6).

**Figure 7:**
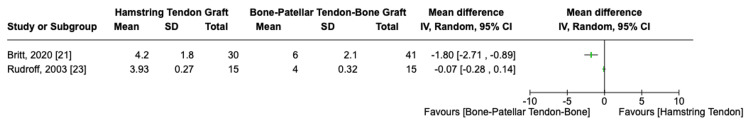
Tegner activity score forest plot

A meta-analysis was not performed for the Tegner activity score due to substantial heterogeneity between the two studies (I² = 92%). Britt et al. reported a statistically significant difference in favor of the BPTB group (p = 0.004) in their study [21], while Rudroff’s study demonstrated no significant difference between the graft types [23]. These findings are illustrated in Figure 7.

Donor-Site Morbidity

Guglielmetti et al. was the only study to report donor-site morbidity [6]. The study found a significantly lower incidence of anterior knee pain in the HT group, with seven out of 31 players (22.6%) affected, compared to 15 out of 31 players (48.4%) in the BPTB group (p = 0.03). The incidence of patellar tendonitis was also lower in the HT group (9.7% vs. 25.8%), although this difference did not reach statistical significance (p = 0.10). Conversely, hamstring tendonitis was more common in the HT group (22.6% vs. 3.2%), but this difference was also not statistically significant (p = 0.05). These findings are illustrated in Table 3.

**Table 3:** Donor-site morbidity effect direction table for HT Effect direction: upward arrow (↑) = positive effect; downward arrow (↓) = negative effect HT, hamstring tendon; RCT, randomized controlled trial

Study	Study design	Anterior knee pain	Patellar tendonitis	Hamstring tendonitis
Guglielmetti et al. (2021) [6]	RCT	↓	↓	↑

Postoperative Knee Stability Outcomes

Postoperative knee stability was assessed only in Guglielmetti et al. using the Lachman and anterior drawer tests at two years post-surgery to assess stability [6]. The study found no statistically significant differences between the graft types, with p-values of 0.94 and 0.71 for the Lachman and anterior drawer tests, respectively.

Risk of Bias Assessment

**Figure 8:**
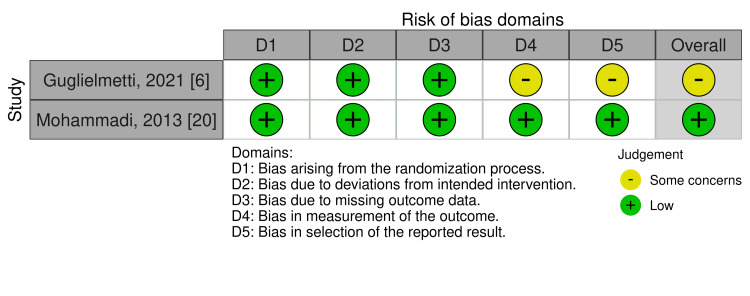
Risk of bias summary of RCTs using the RoB2 tool RCT, randomized controlled trial; RoB2, Risk of Bias 2

**Figure 9:**
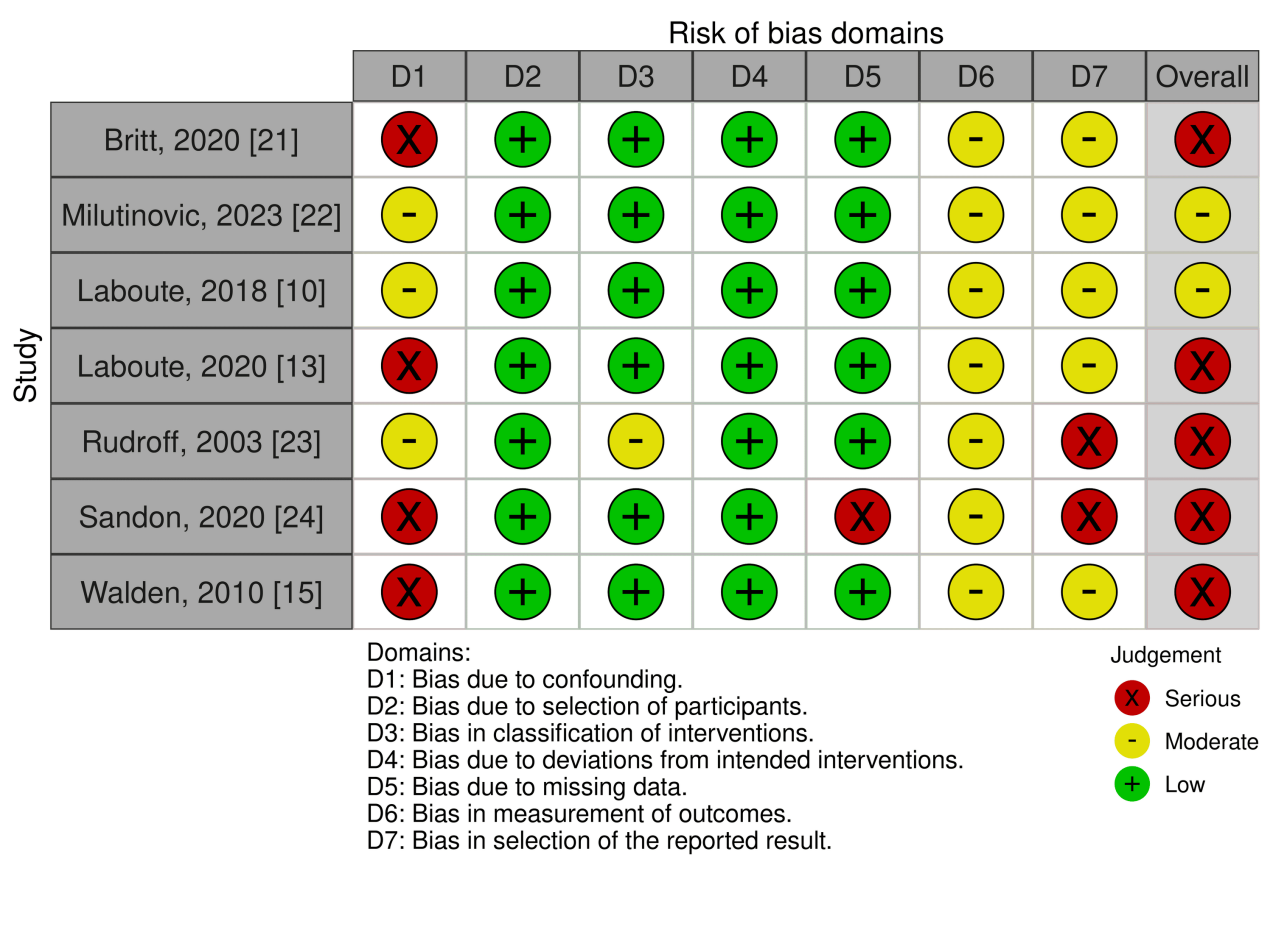
Risk of bias summary of observational studies using the ROBINS-I V2 tool ROBINS-I V2, Risk of Bias in Non-Randomized Studies of Interventions Version 2

Among the two RCTs included, the overall risk of bias was assessed as “some concerns” in one study [6] and “low” in the other [20], according to the RoB2 tool (Figure 8). For the observational studies, the risk of bias was rated as “serious” in five studies [13,15,21,23,24] and “moderate” in two studies [10,22], based on the ROBINS-I V2 tool (Figure 9).

Discussion

In this systematic review and meta-analysis, we evaluated the clinical outcomes of ACL reconstruction using HT vs. BPTB autografts in football players. Across five studies included in the meta-analysis, we found no statistically significant difference in graft failure rates between the two graft types. This finding is in line with previous high-quality meta-analyses in broader population groups, which similarly demonstrated no significant difference in failure rates between HT and BPTB grafts [11,12]. Although a trend favoring lower failure rates with BPTB grafts has been described, the overall evidence suggests comparable graft survival between the two options.

In the present review, the absence of significant differences was further supported by individual studies. The only included RCT by Guglielmetti et al. reported equal graft failure rates between HT and BPTB autografts [6], and Sandon et al., which provided the longest follow-up at 10 years, demonstrated similar long-term graft survivorship in both groups [24]. The results of this review therefore indicate that, amongst football players, graft choice does not significantly influence graft survivorship in the long term. These findings contrast with large-scale registry data from Scandinavian cohorts that identified a higher risk of revision following HT reconstruction, particularly in younger athletes [8-10]. Such differences may reflect variations in surgical technique, graft fixation, and rehabilitation protocols rather than intrinsic differences in graft performance.

Return to sport is a key outcome for football players. Two studies [6,21] contributed to the pooled analysis of return rates and found no significant difference between the graft types, with both associated with return-to-play rates exceeding 75%. Walden et al. reported shorter time to return to both training and match play in the HT group, although the difference was not statistically significant [15]. This finding aligns with that of Laboute et al. [13], who observed that athletes with an HT graft tended to return to competition earlier than those with a BPTB graft (8.8 months vs. 9.4 months, respectively). While not statistically significant, these results may suggest that HT grafts could be a more suitable option for football players seeking a shorter rehabilitation period: for instance, those aiming to return in time for an upcoming competitive season or tournament.

Isokinetic muscle strength recovery differed according to graft choice, though the available evidence was limited. Meta-analysis demonstrated greater postoperative extensor muscle strength in the HT group, which is biomechanically plausible given preservation of the patellar tendon. However, this finding should be interpreted with caution, as it was based on only two studies and was heavily weighted toward a single trial, with the study by Mohammadi et al. contributing the majority of the pooled estimate [20]. As a result, the robustness and generalizability of this finding are limited. In contrast, BPTB grafts were associated with superior long-term flexor strength in one study [23], while early postoperative assessments demonstrated comparable flexor strength between grafts [20,22]. Collectively, these findings suggest that graft choice may differentially affect specific muscle groups, but firm conclusions cannot be drawn based on the current evidence.

PROMs, including Lysholm and Tegner scores, showed no consistent differences between grafts. While the study by Britt et al. [21] reported a statistically significant difference in postoperative Tegner scores favoring BPTB grafts, this finding was not replicated in the other study reporting this outcome [23]. It is important to note that Britt et al.’s study focused exclusively on skeletally mature adolescent female football players, which may limit the generalizability of the result. Knee stability outcomes were also comparable between graft types, with the single study assessing this outcome reporting no significant differences [6], supporting the view that both grafts can provide adequate functional stability when modern fixation techniques are used [12].

Donor-site morbidity emerged as the most clinically relevant difference between graft types in this review. In the only study assessing this outcome, HT autografts were associated with significantly less anterior knee pain and lower rates of patellar tendonitis, consistent with previous literature [11,12]. Although hamstring tendonitis was more frequently reported in the HT group, this did not reach statistical significance. These results suggest that HT grafts may be more suitable for football players where anterior knee discomfort could compromise performance, such as goalkeepers, who heavily rely on kneeling, diving, and ground contact.

Several limitations should be considered when interpreting these findings. Although five studies contributed to the primary outcome, the number of studies reporting each secondary outcome was limited, reducing statistical power and precluding subgroup analyses. Most included studies were observational in design, with moderate to serious risk of bias, and only two were RCTs. In addition, heterogeneity in follow-up duration (ranging from six months to 10 years), rehabilitation protocols, and participant characteristics further limited comparability across studies, particularly for isokinetic strength and PROMs. Few studies stratified outcomes by sex or level of competition, both of which may be relevant in football-specific populations. Finally, exclusion of non-English language studies may have introduced language bias.

## Conclusions

This systematic review and meta-analysis demonstrates, with robust and consistent evidence, that graft failure rates and return-to-sport outcomes do not differ significantly between HT and BPTB autografts in football players undergoing ACL reconstruction. These findings are supported by a well-powered meta-analysis with low heterogeneity and represent the most reliable conclusions of the present study. In contrast, evidence regarding secondary outcomes, including isokinetic muscle strength recovery, donor-site morbidity, PROMs, and knee stability, is limited and should be interpreted with caution. These outcomes were reported by only one or two studies, often with small sample sizes and methodological heterogeneity, precluding firm conclusions.

While some trends were observed, particularly regarding extensor strength and anterior knee pain, these findings remain preliminary and hypothesis-generating rather than definitive. Based on the current evidence, neither graft demonstrates clear overall superiority in football-specific populations. However, meaningful conclusions beyond graft survival and return to sport are constrained by the limited and heterogeneous nature of available data. Further high-quality, adequately powered, football-specific randomized trials with standardized outcome reporting are required to better inform graft selection and optimize clinical decision-making in this population.

## Figures and Tables

**Figure 10:**
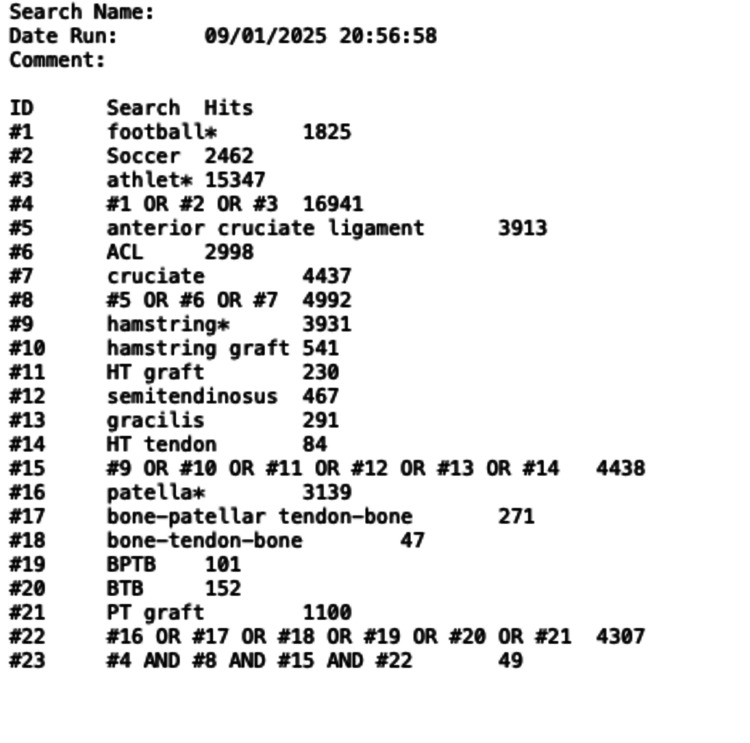
Cochrane search strategy

## Appendices

Appendix A 

**Table 4:** MEDLINE search strategy

#	Query	Results from January 20, 2025
1	exp Soccer/	11,252
2	exp Athletes/	23,488
3	exp Athletic injuries/ or football*.mp.	46,859
4	1 or 2 or 3	69,837
5	exp Anterior Cruciate Ligament/	12,871
6	exp Anterior Cruciate Ligament Reconstruction/ or exp Anterior Cruciate Ligament Injuries/ or ACL.mp.	29,042
7	5 or 6	31,189
8	exp Hamstring Tendons/	864
9	hamstring graft.mp.	571
10	HT graft.mp.	51
11	semitendinosus.mp.	4,718
12	gracilis.mp.	9,063
13	8 or 9 or 10 or 11 or 12	13,701
14	patella*.mp.	28,706
15	exp Patellar Ligament/	3,145
16	patellar tendon.mp.	6,519
17	patellar graft.mp.	30
18	exp Bone-Patellar Tendon-Bone Grafting/	589
19	BPTB.mp.	520
20	BTB.mp.	4,561
21	PT graft.mp.	29
22	14 or 15 or 16 or 17 or 18 or 19 or 20 or 21	33,045
23	4 and 7 and 13 and 22	48

Appendix B  

Appendix C 

**Table 5:** PubMed search strategy

Search number	Query	Search details	Results	Time
9	((((((((football*) OR (soccer[MeSH Terms])) OR (athletes[MeSH Terms])) OR ("football players")) OR (football athletes)) OR ("soccer players")) AND ((((((((((ACL) OR ("ACL injury")) OR ("ACL tear")) OR (cruciate*)) OR (anterior cruciate ligament*)) OR (anterior cruciate ligament[MeSH Terms])) OR (anterior cruciate ligaments[MeSH Terms])) OR (anterior cruciate ligament injuries[MeSH Terms])) AND (((reconstruction) OR (repair)) OR (surgery))) OR (anterior cruciate ligament reconstruction[MeSH Terms]))) AND (((((((hamstring tendon) OR (hamstring graft)) OR (hamstring*)) OR (HT graft)) OR (semitendinosus)) OR (gracilis)) OR (hamstring tendons[MeSH Terms]))) AND (((((((((((patellar tendon) OR (patella*)) OR (patellar graft)) OR (bone-patellar tendon-bone)) OR (bone-tendon-bone)) OR (BPTB)) OR (BTB)) OR (PT graft)) OR (bone patellar tendon bone graft[MeSH Terms])) OR (bone patellar tendon bone grafts[MeSH Terms])) OR (patellar ligament[MeSH Terms]))	("football*"[All Fields] OR "soccer"[MeSH Terms] OR "athletes"[MeSH Terms] OR "football players"[All Fields] OR (("football"[MeSH Terms] OR "football"[All Fields] OR "football s"[All Fields] OR "footballer"[All Fields] OR "footballer s"[All Fields] OR "footballers"[All Fields] OR "footballs"[All Fields]) AND ("athlete s"[All Fields] OR "athletes"[MeSH Terms] OR "athletes"[All Fields] OR "athlete"[All Fields] OR "athletically"[All Fields] OR "athlets"[All Fields] OR "sports"[MeSH Terms] OR "sports"[All Fields] OR "athletic"[All Fields] OR "athletics"[All Fields])) OR "soccer players"[All Fields]) AND ((("anterior cruciate ligament"[MeSH Terms] OR ("anterior"[All Fields] AND "cruciate"[All Fields] AND "ligament"[All Fields]) OR "anterior cruciate ligament"[All Fields] OR "acl"[All Fields] OR "ACL injury"[All Fields] OR "ACL tear"[All Fields] OR "cruciate*"[All Fields] OR (("anterior"[All Fields] OR "anteriores"[All Fields] OR "anteriorization"[All Fields] OR "anteriorized"[All Fields] OR "anteriors"[All Fields]) AND ("cruciate"[All Fields] OR "cruciates"[All Fields]) AND "ligament*"[All Fields]) OR "anterior cruciate ligament"[MeSH Terms] OR "anterior cruciate ligament"[MeSH Terms] OR "anterior cruciate ligament injuries"[MeSH Terms]) AND ("plastic surgery procedures"[MeSH Terms] OR ("plastic"[All Fields] AND "surgery"[All Fields] AND "procedures"[All Fields]) OR "plastic surgery procedures"[All Fields] OR "reconstruction"[All Fields] OR "reconstructions"[All Fields] OR "reconstruct"[All Fields] OR "reconstructability"[All Fields] OR "reconstructable"[All Fields] OR "reconstructed"[All Fields] OR "reconstructible"[All Fields] OR "reconstructing"[All Fields] OR "reconstructional"[All Fields] OR "reconstructive"[All Fields] OR "reconstructs"[All Fields] OR ("repairability"[All Fields] OR "repairable"[All Fields] OR "repaire"[All Fields] OR "repaired"[All Fields] OR "repairment"[All Fields] OR "wound healing"[MeSH Terms] OR ("wound"[All Fields] AND "healing"[All Fields]) OR "wound healing"[All Fields] OR "repair"[All Fields] OR "repairing"[All Fields] OR "repairs"[All Fields]) OR ("surgery"[MeSH Subheading] OR "surgery"[All Fields] OR "surgical procedures, operative"[MeSH Terms] OR ("surgical"[All Fields] AND "procedures"[All Fields] AND "operative"[All Fields]) OR "operative surgical procedures"[All Fields] OR "general surgery"[MeSH Terms] OR ("general"[All Fields] AND "surgery"[All Fields]) OR "general surgery"[All Fields] OR "surgery s"[All Fields] OR "surgerys"[All Fields] OR "surgeries"[All Fields]))) OR "anterior cruciate ligament reconstruction"[MeSH Terms]) AND ("hamstring tendons"[MeSH Terms] OR ("hamstring"[All Fields] AND "tendons"[All Fields]) OR "hamstring tendons"[All Fields] OR ("hamstring"[All Fields] AND "tendon"[All Fields]) OR "hamstring tendon"[All Fields] OR (("hamstring muscles"[MeSH Terms] OR ("hamstring"[All Fields] AND "muscles"[All Fields]) OR "hamstring muscles"[All Fields] OR "hamstring"[All Fields] OR "hamstrings"[All Fields]) AND ("graft s"[All Fields] OR "grafted"[All Fields] OR "graftings"[All Fields] OR "transplantation"[MeSH Subheading] OR "transplantation"[All Fields] OR "grafting"[All Fields] OR "transplantation"[MeSH Terms] OR "grafts"[All Fields] OR "transplants"[MeSH Terms] OR "transplants"[All Fields] OR "graft"[All Fields])) OR "hamstring*"[All Fields] OR (("ht acm conf hypertext soc media"[Journal] OR "ht"[All Fields]) AND ("graft s"[All Fields] OR "grafted"[All Fields] OR "graftings"[All Fields] OR "transplantation"[MeSH Subheading] OR "transplantation"[All Fields] OR "grafting"[All Fields] OR "transplantation"[MeSH Terms] OR "grafts"[All Fields] OR "transplants"[MeSH Terms] OR "transplants"[All Fields] OR "graft"[All Fields])) OR ("hamstring muscles"[MeSH Terms] OR ("hamstring"[All Fields] AND "muscles"[All Fields]) OR "hamstring muscles"[All Fields] OR "semitendinosus"[All Fields]) OR ("gracilis muscle"[MeSH Terms] OR ("gracilis"[All Fields] AND "muscle"[All Fields]) OR "gracilis muscle"[All Fields] OR "gracilis"[All Fields]) OR "hamstring tendons"[MeSH Terms]) AND ("patellar ligament"[MeSH Terms] OR ("patellar"[All Fields] AND "ligament"[All Fields]) OR "patellar ligament"[All Fields] OR ("patellar"[All Fields] AND "tendon"[All Fields]) OR "patellar tendon"[All Fields] OR "patella*"[All Fields] OR (("patella"[MeSH Terms] OR "patella"[All Fields] OR "patellar"[All Fields]) AND ("graft s"[All Fields] OR "grafted"[All Fields] OR "graftings"[All Fields] OR "transplantation"[MeSH Subheading] OR "transplantation"[All Fields] OR "grafting"[All Fields] OR "transplantation"[MeSH Terms] OR "grafts"[All Fields] OR "transplants"[MeSH Terms] OR "transplants"[All Fields] OR "graft"[All Fields])) OR ("bone-patellar"[All Fields] AND "tendon-bone"[All Fields]) OR "bone-tendon-bone"[All Fields] OR "BPTB"[All Fields] OR "BTB"[All Fields] OR (("perspect terror"[Journal] OR "pt"[All Fields]) AND ("graft s"[All Fields] OR "grafted"[All Fields] OR "graftings"[All Fields] OR "transplantation"[MeSH Subheading] OR "transplantation"[All Fields] OR "grafting"[All Fields] OR "transplantation"[MeSH Terms] OR "grafts"[All Fields] OR "transplants"[MeSH Terms] OR "transplants"[All Fields] OR "graft"[All Fields])) OR "bone patellar tendon bone grafts"[MeSH Terms] OR "bone patellar tendon bone grafts"[MeSH Terms] OR "patellar ligament"[MeSH Terms])	61	16:20:53
8	((((((((((patellar tendon) OR (patella*)) OR (patellar graft)) OR (bone-patellar tendon-bone)) OR (bone-tendon-bone)) OR (BPTB)) OR (BTB)) OR (PT graft)) OR (bone patellar tendon bone graft[MeSH Terms])) OR (bone patellar tendon bone grafts[MeSH Terms])) OR (patellar ligament[MeSH Terms])	"patellar ligament"[MeSH Terms] OR ("patellar"[All Fields] AND "ligament"[All Fields]) OR "patellar ligament"[All Fields] OR ("patellar"[All Fields] AND "tendon"[All Fields]) OR "patellar tendon"[All Fields] OR "patella*"[All Fields] OR (("patella"[MeSH Terms] OR "patella"[All Fields] OR "patellar"[All Fields]) AND ("graft s"[All Fields] OR "grafted"[All Fields] OR "graftings"[All Fields] OR "transplantation"[MeSH Subheading] OR "transplantation"[All Fields] OR "grafting"[All Fields] OR "transplantation"[MeSH Terms] OR "grafts"[All Fields] OR "transplants"[MeSH Terms] OR "transplants"[All Fields] OR "graft"[All Fields])) OR ("bone-patellar"[All Fields] AND "tendon-bone"[All Fields]) OR "bone-tendon-bone"[All Fields] OR "BPTB"[All Fields] OR "BTB"[All Fields] OR (("perspect terror"[Journal] OR "pt"[All Fields]) AND ("graft s"[All Fields] OR "grafted"[All Fields] OR "graftings"[All Fields] OR "transplantation"[MeSH Subheading] OR "transplantation"[All Fields] OR "grafting"[All Fields] OR "transplantation"[MeSH Terms] OR "grafts"[All Fields] OR "transplants"[MeSH Terms] OR "transplants"[All Fields] OR "graft"[All Fields])) OR "bone patellar tendon bone grafts"[MeSH Terms] OR "bone patellar tendon bone grafts"[MeSH Terms] OR "patellar ligament"[MeSH Terms]	37,000	16:20:26
7	((((((hamstring tendon) OR (hamstring graft)) OR (hamstring*)) OR (HT graft)) OR (semitendinosus)) OR (gracilis)) OR (hamstring tendons[MeSH Terms])	"hamstring tendons"[MeSH Terms] OR ("hamstring"[All Fields] AND "tendons"[All Fields]) OR "hamstring tendons"[All Fields] OR ("hamstring"[All Fields] AND "tendon"[All Fields]) OR "hamstring tendon"[All Fields] OR (("hamstring muscles"[MeSH Terms] OR ("hamstring"[All Fields] AND "muscles"[All Fields]) OR "hamstring muscles"[All Fields] OR "hamstring"[All Fields] OR "hamstrings"[All Fields]) AND ("graft s"[All Fields] OR "grafted"[All Fields] OR "graftings"[All Fields] OR "transplantation"[MeSH Subheading] OR "transplantation"[All Fields] OR "grafting"[All Fields] OR "transplantation"[MeSH Terms] OR "grafts"[All Fields] OR "transplants"[MeSH Terms] OR "transplants"[All Fields] OR "graft"[All Fields])) OR "hamstring*"[All Fields] OR (("ht acm conf hypertext soc media"[Journal] OR "ht"[All Fields]) AND ("graft s"[All Fields] OR "grafted"[All Fields] OR "graftings"[All Fields] OR "transplantation"[MeSH Subheading] OR "transplantation"[All Fields] OR "grafting"[All Fields] OR "transplantation"[MeSH Terms] OR "grafts"[All Fields] OR "transplants"[MeSH Terms] OR "transplants"[All Fields] OR "graft"[All Fields])) OR ("hamstring muscles"[MeSH Terms] OR ("hamstring"[All Fields] AND "muscles"[All Fields]) OR "hamstring muscles"[All Fields] OR "semitendinosus"[All Fields]) OR ("gracilis muscle"[MeSH Terms] OR ("gracilis"[All Fields] AND "muscle"[All Fields]) OR "gracilis muscle"[All Fields] OR "gracilis"[All Fields]) OR "hamstring tendons"[MeSH Terms]	26,364	16:18:53
6	(((((((((ACL) OR ("ACL injury")) OR ("ACL tear")) OR (cruciate*)) OR (anterior cruciate ligament*)) OR (anterior cruciate ligament[MeSH Terms])) OR (anterior cruciate ligaments[MeSH Terms])) OR (anterior cruciate ligament injuries[MeSH Terms])) AND (((reconstruction) OR (repair)) OR (surgery))) OR (anterior cruciate ligament reconstruction[MeSH Terms])	(("anterior cruciate ligament"[MeSH Terms] OR ("anterior"[All Fields] AND "cruciate"[All Fields] AND "ligament"[All Fields]) OR "anterior cruciate ligament"[All Fields] OR "acl"[All Fields] OR "ACL injury"[All Fields] OR "ACL tear"[All Fields] OR "cruciate*"[All Fields] OR (("anterior"[All Fields] OR "anteriores"[All Fields] OR "anteriorization"[All Fields] OR "anteriorized"[All Fields] OR "anteriors"[All Fields]) AND ("cruciate"[All Fields] OR "cruciates"[All Fields]) AND "ligament*"[All Fields]) OR "anterior cruciate ligament"[MeSH Terms] OR "anterior cruciate ligament"[MeSH Terms] OR "anterior cruciate ligament injuries"[MeSH Terms]) AND ("plastic surgery procedures"[MeSH Terms] OR ("plastic"[All Fields] AND "surgery"[All Fields] AND "procedures"[All Fields]) OR "plastic surgery procedures"[All Fields] OR "reconstruction"[All Fields] OR "reconstructions"[All Fields] OR "reconstruct"[All Fields] OR "reconstructability"[All Fields] OR "reconstructable"[All Fields] OR "reconstructed"[All Fields] OR "reconstructible"[All Fields] OR "reconstructing"[All Fields] OR "reconstructional"[All Fields] OR "reconstructive"[All Fields] OR "reconstructs"[All Fields] OR ("repairability"[All Fields] OR "repairable"[All Fields] OR "repaire"[All Fields] OR "repaired"[All Fields] OR "repairment"[All Fields] OR "wound healing"[MeSH Terms] OR ("wound"[All Fields] AND "healing"[All Fields]) OR "wound healing"[All Fields] OR "repair"[All Fields] OR "repairing"[All Fields] OR "repairs"[All Fields]) OR ("surgery"[MeSH Subheading] OR "surgery"[All Fields] OR "surgical procedures, operative"[MeSH Terms] OR ("surgical"[All Fields] AND "procedures"[All Fields] AND "operative"[All Fields]) OR "operative surgical procedures"[All Fields] OR "general surgery"[MeSH Terms] OR ("general"[All Fields] AND "surgery"[All Fields]) OR "general surgery"[All Fields] OR "surgery s"[All Fields] OR "surgerys"[All Fields] OR "surgeries"[All Fields]))) OR "anterior cruciate ligament reconstruction"[MeSH Terms]	31,470	16:17:46
5	anterior cruciate ligament reconstruction[MeSH Terms]	"anterior cruciate ligament reconstruction"[MeSH Terms]	8,229	16:17:30
4	((((((((ACL) OR ("ACL injury")) OR ("ACL tear")) OR (cruciate*)) OR (anterior cruciate ligament*)) OR (anterior cruciate ligament[MeSH Terms])) OR (anterior cruciate ligaments[MeSH Terms])) OR (anterior cruciate ligament injuries[MeSH Terms])) AND (((reconstruction) OR (repair)) OR (surgery))	("anterior cruciate ligament"[MeSH Terms] OR ("anterior"[All Fields] AND "cruciate"[All Fields] AND "ligament"[All Fields]) OR "anterior cruciate ligament"[All Fields] OR "acl"[All Fields] OR "ACL injury"[All Fields] OR "ACL tear"[All Fields] OR "cruciate*"[All Fields] OR (("anterior"[All Fields] OR "anteriores"[All Fields] OR "anteriorization"[All Fields] OR "anteriorized"[All Fields] OR "anteriors"[All Fields]) AND ("cruciate"[All Fields] OR "cruciates"[All Fields]) AND "ligament*"[All Fields]) OR "anterior cruciate ligament"[MeSH Terms] OR "anterior cruciate ligament"[MeSH Terms] OR "anterior cruciate ligament injuries"[MeSH Terms]) AND ("plastic surgery procedures"[MeSH Terms] OR ("plastic"[All Fields] AND "surgery"[All Fields] AND "procedures"[All Fields]) OR "plastic surgery procedures"[All Fields] OR "reconstruction"[All Fields] OR "reconstructions"[All Fields] OR "reconstruct"[All Fields] OR "reconstructability"[All Fields] OR "reconstructable"[All Fields] OR "reconstructed"[All Fields] OR "reconstructible"[All Fields] OR "reconstructing"[All Fields] OR "reconstructional"[All Fields] OR "reconstructive"[All Fields] OR "reconstructs"[All Fields] OR ("repairability"[All Fields] OR "repairable"[All Fields] OR "repaire"[All Fields] OR "repaired"[All Fields] OR "repairment"[All Fields] OR "wound healing"[MeSH Terms] OR ("wound"[All Fields] AND "healing"[All Fields]) OR "wound healing"[All Fields] OR "repair"[All Fields] OR "repairing"[All Fields] OR "repairs"[All Fields]) OR ("surgery"[MeSH Subheading] OR "surgery"[All Fields] OR "surgical procedures, operative"[MeSH Terms] OR ("surgical"[All Fields] AND "procedures"[All Fields] AND "operative"[All Fields]) OR "operative surgical procedures"[All Fields] OR "general surgery"[MeSH Terms] OR ("general"[All Fields] AND "surgery"[All Fields]) OR "general surgery"[All Fields] OR "surgery s"[All Fields] OR "surgerys"[All Fields] OR "surgeries"[All Fields]))	31,442	16:17:04
3	((reconstruction) OR (repair)) OR (surgery)	"plastic surgery procedures"[MeSH Terms] OR ("plastic"[All Fields] AND "surgery"[All Fields] AND "procedures"[All Fields]) OR "plastic surgery procedures"[All Fields] OR "reconstruction"[All Fields] OR "reconstructions"[All Fields] OR "reconstruct"[All Fields] OR "reconstructability"[All Fields] OR "reconstructable"[All Fields] OR "reconstructed"[All Fields] OR "reconstructible"[All Fields] OR "reconstructing"[All Fields] OR "reconstructional"[All Fields] OR "reconstructive"[All Fields] OR "reconstructs"[All Fields] OR ("repairability"[All Fields] OR "repairable"[All Fields] OR "repaire"[All Fields] OR "repaired"[All Fields] OR "repairment"[All Fields] OR "wound healing"[MeSH Terms] OR ("wound"[All Fields] AND "healing"[All Fields]) OR "wound healing"[All Fields] OR "repair"[All Fields] OR "repairing"[All Fields] OR "repairs"[All Fields]) OR ("surgery"[MeSH Subheading] OR "surgery"[All Fields] OR "surgical procedures, operative"[MeSH Terms] OR ("surgical"[All Fields] AND "procedures"[All Fields] AND "operative"[All Fields]) OR "operative surgical procedures"[All Fields] OR "general surgery"[MeSH Terms] OR ("general"[All Fields] AND "surgery"[All Fields]) OR "general surgery"[All Fields] OR "surgery s"[All Fields] OR "surgerys"[All Fields] OR "surgeries"[All Fields])	6,490,445	16:16:50
2	(((((((ACL) OR ("ACL injury")) OR ("ACL tear")) OR (cruciate*)) OR (anterior cruciate ligament*)) OR (anterior cruciate ligament[MeSH Terms])) OR (anterior cruciate ligaments[MeSH Terms])) OR (anterior cruciate ligament injuries[MeSH Terms])	"anterior cruciate ligament"[MeSH Terms] OR ("anterior"[All Fields] AND "cruciate"[All Fields] AND "ligament"[All Fields]) OR "anterior cruciate ligament"[All Fields] OR "acl"[All Fields] OR "ACL injury"[All Fields] OR "ACL tear"[All Fields] OR "cruciate*"[All Fields] OR (("anterior"[All Fields] OR "anteriores"[All Fields] OR "anteriorization"[All Fields] OR "anteriorized"[All Fields] OR "anteriors"[All Fields]) AND ("cruciate"[All Fields] OR "cruciates"[All Fields]) AND "ligament*"[All Fields]) OR "anterior cruciate ligament"[MeSH Terms] OR "anterior cruciate ligament"[MeSH Terms] OR "anterior cruciate ligament injuries"[MeSH Terms]	45,690	16:16:21
1	(((((football*) OR (soccer[MeSH Terms])) OR (athletes[MeSH Terms])) OR ("football players")) OR (football athletes)) OR ("soccer players")	"football*"[All Fields] OR "soccer"[MeSH Terms] OR "athletes"[MeSH Terms] OR "football players"[All Fields] OR (("football"[MeSH Terms] OR "football"[All Fields] OR "football s"[All Fields] OR "footballer"[All Fields] OR "footballer s"[All Fields] OR "footballers"[All Fields] OR "footballs"[All Fields]) AND ("athlete s"[All Fields] OR "athletes"[MeSH Terms] OR "athletes"[All Fields] OR "athlete"[All Fields] OR "athletically"[All Fields] OR "athlets"[All Fields] OR "sports"[MeSH Terms] OR "sports"[All Fields] OR "athletic"[All Fields] OR "athletics"[All Fields])) OR "soccer players"[All Fields]	47,976	16:14:41
